# The impact of dispensing fees on compliance with opioid substitution therapy: a mixed methods study

**DOI:** 10.1186/1747-597X-9-32

**Published:** 2014-08-10

**Authors:** Alexandra Shepherd, Bianca Perrella, Hendrika Laetitia Hattingh

**Affiliations:** 1 Curtin Health, Curtin University, Bentley, Perth, Western Australia 6845, Australia

## Abstract

**Background:**

Opioid substitution therapy (OST) programs involve the dispensing of OST medicines to patients to address their dependence on heroin and/or other opioid substances. OST medicines are subsidised by the Australian government but patients need to pay the dispensing fees. This study explored opinions from OST patients and stakeholders about the potential impact of dispensing fees on compliance and OST program retention. Current and past experiences and the potential impact of OST dispensing fees were evaluated.

**Methods:**

Mixed methodology was used to obtain data from OST patients and stakeholders. This involved 1) interviews with OST stakeholders, 2) a focus group of OST patients and 3) surveys of OST patients in Perth, Australia, between June and August 2013.

**Results:**

The majority of the eight stakeholders declared cost as the factor mostly impacting on OST compliance. Almost all of the stakeholders commented that there was a positive correlation between time on the OST program and success in terms of relapse. Most stakeholders advocated for OST fees to contribute towards the Pharmaceutical Benefits Scheme Safety Net, and for fee subsidy. Focus group themes supported stakeholder interview findings. A total of 138 surveys were completed. Survey analysis illustrated a strong correlation between patient debt and impacted lifestyle: 82.4% (p < 0.001, Chi-square test) of the 138 survey participants stated that dispensing fees impacted significantly on patients’ finances and lifestyle, specifically those patients with major debt. The cost of dispensing fees was identified by 46.3% (64/138) of survey participants as the biggest impacting factor on patient success. Logistic regression models showed that the cost of dispensing fees was also found to significantly influence both the occurrence of debt (57.7%, p < 0.0001) and lifestyle difficulties (80.0%, p = 0.0004).

**Conclusion:**

Findings provided insight into OST patients’ financial difficulties with data suggesting that dispensing fees are likely to have a negative impact on OST patients’ compliance with therapy, retention in the OST program and lifestyle. Government sponsorship of the OST dispensing fees should be considered as sponsorship would potentially increase the retention rates of income-poor OST program recipients.

## Introduction

Opioid substitution therapy (OST) programs are successful at addressing dependence on heroin and other opioid substances [1,2]. These programs involve the assessment and treatment of people with an opioid addiction and the dispensing of OST medicines to address their dependence on heroin and/or other opioid substances. This, in turn, reduces the negative behaviours, health issues, physiological issues and crime associated with opioid misuse [3,4]. OST has been proven cost-effective [2,5,6] for governments due to the reduction of illicit drug use costs to the health system [1,7,8] and decreased crime rate and other indirect costs to society [9,10]. The primary aim of OST is for patients to decrease and eventually cease their opioid misuse and improve their quality of life [11]. The positive outcomes observed from OST include decreased risk of injecting infections, illnesses and fatalities [9,12] and increased ability to perform daily tasks as compared to when using illicit heroin [4].

There are three OST medicines available in Australia and all three should be administered at a pharmacy under a pharmacist’s supervision, unless take-away doses have been authorised. The most commonly used medicine is methadone which needs to be taken daily. Buprenorphine (branded as Subutex®) and buprenorphine with naloxone (branded as Suboxone®) have varied dosing requirements with frequency ranging between daily to every third day [13]. All three OST medicines are subsidised by the Australian government through Section 100 of the National Health Act 1953 (Cth) and are included in the Pharmaceutical Benefits Scheme (PBS) [14,15]. However, the dispensing fees associated with supplying the medicines have to be paid by patients and do not contribute towards the Safety Net Scheme [12,16], which is a scheme that provides financial assistance to patients with substantial pharmaceutical expenses [17]. There is no set dispensing fee charged to OST patients and there are large price disparities across community pharmacies in the various Australian jurisdictions [12]. Previous research indicated that OST fees ranged between $18 and $56 per week [18].

Various approaches need to be followed in the management of opioid addiction and there are many variables that may affect a patient’s success and retention on the OST program. Although financial support towards dispensing fees is only one aspect to encourage patients to be treated and be retained in care financial instability is one concern that is frequently raised [10,18,19]. The demographic of patients on OST is generally in the lower income bracket and it was estimated in 2005 that only 17% of Australian OST patients received their main income from paid work [20]. Income from government support has, however, been reported to be insufficient for patients to buy groceries with some patients having to prioritise their OST fees over buying food on certain occasions [15]. It has also been found that large numbers of patients have acquired significant debt to pharmacies to cover the dispensing fees [12,21-24].

A 2008 Australian study involving 508 OST patients found that 23% of the patients owed money to pharmacies and 32% of the patients could not afford the dispensing fees, which resulted in some patients missing their dose(s) [18]. This study also indicated that patients were not satisfied with the dosing hours, number of venues available to dose, and not having enough take-away doses [18]. A study conducted in the United States of America (USA) evaluated the retention rates of patients on OST within two states after OST treatment subsidisation by Medicaid was introduced [19]. The retention rates increased from 28% to 51% in one state and from 28% to 34% in the other, suggesting that financial aid can positively impact OST retention rates [19]. Another USA study in which patients were provided with incentive vouchers in order to increase program adherence similarly showed increased retention rates [25].

Although there has been some research into the factors influencing OST program retention rates there has been limited research covering OST patients’ finances and the potential impact of dispensing fees on compliance and retention in the OST program[19,26]. This study aimed to obtain opinions and feedback from patients and stakeholders about the impact of dispensing fees on patients’ debt, lifestyles and success or retention in the OST program.

## Methods

This study was conducted in Perth, Western Australia, between June and August 2013. A mixed methodology involving quantitative and qualitative data collection strategies was used to obtain data from OST patients and stakeholders. Three approaches were utilised namely 1) interviews with OST stakeholders, 2) a focus group of OST patients and 3) surveys of OST patients. All information and data collected were de-identified and participants’ identities remained confidential. Ethics approval was obtained from the Curtin University Human Ethics Committee (approval number PH-10-13).

### Stakeholder and focus group interviews

A stakeholder interview guide was developed to gather opinions and perspectives on how OST dispensing fees potentially impacted on patients and whether there could be a correlation between the fees and patient compliance and retention in the program. The interview guide consisted of 10 open-ended questions that covered:

• Whether time on OST correlated with ongoing abstinence from opioids

• OST patient financial aspects: groups of patients who struggle to pay dispensing fees (i.e. single mothers, teenagers), pricing of take-away doses, potential impact of dispensing fees on lifestyle factors, accumulated dispensing fee debt and the potential value of finance workshops to up-skill patients

• Recommendations to improve or regulate the cost of OST dispensing fees.

Stakeholders were purposively selected to ensure participants were knowledgeable about OST medicine supply issues. All stakeholders approached were therefore in regular contact with OST patients and in an ideal position to provide feedback about the program. Potential stakeholder participants were given an information sheet about the study and asked to sign a consent form. Interviews were conducted face-to-face at a time and place that was convenient for the participants.

An open-ended focus group guide was developed to generate discussion with OST patients focusing on:

• The impact of dispensing fees on lifestyle and retention and success with OST

• Potential value of finance and lifestyle workshops to up-skill OST patients

• Recommendations to improve or regulate the cost of OST dispensing fees

Focus group participants were purposively selected by the Western Australia Substance Users Association (WASUA) staff members to involve patients with varying opinions and experiences with the OST program. All participants were given an information sheet about the study and asked to sign a consent form. The focus group was conducted at the WASUA offices.

The interviews and focus group were conducted by members of the research team skilled in interview techniques and the running of a focus group. All stakeholder and focus group interviews were audio-recorded.

### Surveys

A survey consisting of eleven questions was developed to collect some information about OST patients’ status (i.e. relationships, employment and children), duration of OST treatment, factors impacting on the success of treatment, current income level and whether dispensing fees altered their lifestyle or contributed to debt. The survey was designed as a two-page survey as consultation with health professionals involved with OST patients and WASUA staff indicated that the survey had to be short to facilitate participation. The surveys were distributed over a six week period through:

• A specialist OST medical clinic located near the Perth central business district (CBD) that provided services to approximately 500 OST patients

• WASUA, and

• Two community pharmacies located north and south of the Perth CBD. These pharmacies were two of the largest OST dispensing pharmacies in Perth.

These locations were carefully selected to ensure a diverse range of OST patients were surveyed and to reduce any bias towards particular patient demographics. Staff at the various locations were briefed about the study and provided with background information and instructions about eligibility criteria, namely patients on OST at the time of the study who were ≥ 18 years old. The staff made the participant information sheets and surveys available to eligible patients during patient visits, indicating that participation in the survey was voluntary.

### Data analyses

Qualitative analyses: All stakeholder and focus group interviews were manually transcribed. Participants were de-identified and codes were used in the analysis. Data analysis was informed by the general inductive approach [27]: transcript content was read repeatedly by the research team to attain a thorough understanding of topics that emerged from the interviews. A colour-coding scheme was used to identify themes. Topics and themes were developed to capture core messages reported by the participants. Emerged ideas or themes were recorded and supporting quotes documented under each theme. To ensure reliability of the process of analysis all authors reviewed the themes and provided input throughout the data analysis process.

Quantitative analyses: The association between the two primary outcome variables (debt and impact on lifestyle) was assessed using the Chi-square statistic. Logistic regression models were used to identify independent variables which were associated with each of these outcomes. For each outcome, the independent variables included: cost 1) considered to cause a significant impact, 2) cost per fortnight (dispensing fee), and 3) demographic data: single, employed, having dependent children, and time on the OST program. The Likert scale responses (scores: 1 to 5) for the dependent variables were reduced by classifying responses 1,2 as ‘low impact’, and 4,5 as causing significant impact. The middle response indicating ‘sometimes impact’, was excluded from analysis. For each outcome, a ‘backwards elimination’ method was used to reach the optimum model. This method involves fitting all the independent variables to the model initially, then dropping the least significant variable (one at a time) until all variables remaining in the model were significantly associated with the outcome. Results of these regressions were presented as odds ratios, their 95% confidence intervals, and p-values. For all tests, a p-value < 0.05 was taken to indicate a statistically significant association. Analyses were performed using the SPSS version 21 statistical software.

## Results

### Stakeholder and focus group interviews

Stakeholder participants were:

• Three OST support workers (IP1, IP2 and IP3)

• A Community Program for Opioid Pharmacotherapy (CPOP) administrator from the Western Australian Health Department (IP4)

• A CPOP manager (IP5)

• A CPOP co-ordinator (IP6)

• A CPOP prescriber who treated large numbers of OST patients (IP8)

• A community pharmacist manager from a pharmacy that provided OST dispensing services to large numbers of OST patients (IP7)

Stakeholder interviews ranged between 29 to 56 minutes. The focus group of forty minutes involved four OST patients that varied in age, gender, and time on the OST program (between three and 20 years).

Four themes emerged from the stakeholder interviews and focus group data. Emerged themes were recorded and supporting quotes documented under each theme.

#### OST cost and patient drop-out rates

When stakeholder interview participants were asked to comment on the most common factors causing patients to prematurely drop out of the OST program, the majority of stakeholders (5/8) declared cost as the factor mostly impacting on OST compliance. Table 1 provides a summary of the drop-out factors with selected quotes.

**Table 1:** Interview data of the factors influencing patient drop-outs from OST program with selected quotes to support themes

**Themes**	**Selected quotes**
Financial burden	*“All of the money before was coming from crime and they were able to pay hundreds of dollars for heroin. When patients come on methadone they usually don’t have a lot of resources… all would be in debt… credit card debt, debt to drug dealer, debt to family/friends, and ordinary sorts of debt”.* (IP8)
Relapse	*“…patients may not be able to go to the movies or take partner out for dinner because that would mean that next week they won’t be able to afford their dose… bored sitting at home, not able to go out to gym or yoga or movies… left with the option of sitting at home and watching TV. This leads to relapse because they have nothing else to do”.* (IP1)
Lack of support	*“ …people are put on the program without sufficient support and go on without being aware that it is a long term thing”* (IP2)
Insufficient take-away doses - eg. mine workers	*“I think that patients should be able to get more take away doses… be able to self-manage”* (IP1)
Pharmacy discrimination	*“They are treated differently to the other customers. It happens all the time”* (IP3).
Limiting daily life	*“… can be debilitating to be on the program… I travel interstate a lot for work- if the organisation was not aware of my position I could have not held that position down” *(IP2)
	*“liquid handcuffs”* (IP2)
Inadequate preparation	The length of the program … *“may not be entirely understood by the consumer as it may be less important in that moment”* (IP1).
	*“knee jerk reaction – thinking CPOP is going to be the silver bullet for all their problems”* (IP2).
Rural location	*“If someone gets a job up north- may not take the job due to not being able to dose up there”* (IP7)

The local prescriber (IP8) indicated that although factors such as inflexible dosing requirements, discrimination at the pharmacy and travelling restrictions made it difficult for patients to participate in the program, these were not likely to cause patient drop-out as arrangements could be made to overcome most of these barriers. The prescriber suggested that the cost of the dosing and patients’ inability to pay for OST medicines were more likely to contribute towards patient drop-out.

The results obtained from the focus group supported the stakeholder opinions with cost, relapse and inflexibility of the program raised as common factors causing patients drop-outs.

#### OST fees impact on patient lifestyle factors

Stakeholder interview participants were asked how patients’ lifestyles were potentially impacted by having to pay the OST dispensing fees. All participants commented that most, if not all patients entering the OST program, would have financial debt and be at the lowest point in their life. It was also highlighted that many patients were not able to engage in a healthy social life such as participating in activities like going to the movies, doing exercise and fitness classes or going out for dinner or coffee with friends, family or partners.

Another recurring topic was that patients accumulate debt to pharmacies:

“… pharmacies generally allow patients to book up their cost of dosing and then the manager/owner of the store will eventually just cut them off. This can be done at times with no warning …” (IP3).

However, one of the participants (IP7), a pharmacist, commented that 85% of their patients were up-to-date or in advance on their dosing fees. This pharmacy dosed approximately 160 patients and was one of the largest OST dispensing pharmacies in the metropolitan area at the time of the study.

Stakeholder interview participants were asked if they were able to identify any particular demographic groups that were impacted by dispensing fees. A number of different groups were identified, and these included: patients newly released from prison, patients with other medical conditions and expenses, pensioners and the unemployed. The overall consensus was that the majority of patients on the OST program would classify into one of these groups. It was also proposed that the general characteristic of being a previous addict meant that patients’ priorities did not always align with paying fees on time.

The focus group participants identified that being on the OST program impacted significantly on everyday life. For example patients are not able to afford to go on holidays or have a normal social life.

#### Time on OST program and continued opioid abstinence

Stakeholder interview participants were asked to comment on the correlation between time on OST program and patient stability, either when on or off the program. Seven out of the eight interview participants commented that there was a positive correlation between the two, namely the longer a patient stays on the program the better the outcome. Two of the interview participants commented that the stability of a patient is dependent on the individual patient’s situation and support network and that it is therefore difficult to suggest an estimated length of therapy for a patient when commencing with the program. However it was estimated by most participants that the majority of patients would need between six and twelve months on the program to change other facets of their life in order to become stable:

“… six months onwards – can take up to 1-2 years to really get their life back together… employment, fixing their relationships with family and friends, forming new relationships with people outside the drug world…” (IP8).

#### Patient support

Stakeholder interview participants were asked to make suggestions as to what may potentially support patients to better manage their OST fees. Most stakeholders advocated for OST fees to contribute towards the Pharmaceutical Benefits Scheme Safety Net (6/8), and for fee subsidy (5/8). The rationale used by six out of eight participants was that drug addiction should be recognised as a medical condition as is the case with the other conditions covered under the Safety Net Scheme:

“…it is a medical condition. The PBS covers smoking drugs… they are the same concept apart from one drug being legal and the other not …” (IP7).

Other suggestions made were to have a fixed dispensing fee, provide budgeting workshops, allowing patients to skip doses to save money, attend mandatory counselling, offer reimbursements, provide a resource book on where to seek help, and subsidise patients coming out of prison. It was raised by three of the eight interview participants that it was important not to increase barriers to participate in the program:

“… the program has to have a degree of flexibility …” (IP7).

All four focus group members supported the idea of having the PBS Safety Net Scheme apply to OST dispensing fees. The group also expressed the need to have a free dosing clinic that was more readily available for patients that were in need of more financial support.

### Survey results

A total of 138 surveys were completed. Participants included 76 males and 62 females with ages between 21 and 61 years (mean [standard deviation] = 40.0 [9.4]). The data showed a high rate of unemployment (36.1%) amongst the participants with a large portion (52.2%) of the participants being on a low income of less than $500 per fortnight.

#### Dispensing fees and their impact on debt and lifestyle

A cross tabulation was completed to show the percentage of participants who experienced significant impact on lifestyle when in debt due to dispensing fees. Questions 10 and 11 requested information concerning the impact of cost of dispensing fees on lifestyle and whether the fees caused participants to incur any debt. The Chi-square test (Table 2) indicates that there is a strong correlation between debt and impacted lifestyle. Of the participants who were always in debt, the majority also indicated that dispensing fees significantly impacted their lifestyle, while the percentage was significantly lower for those not always in debt (p < 0.001, χ^2^ = 45.3, df = 4).

**Table 2:** Survey data of the impact of dispensing fees on lifestyle and debt of OST patients

		**Lifestyle groups**		
		**No impact (%)**	**Sometimes impacts (%)**	**Significantly impacts (%)**
**Debt groups (%)**	**Never in debt**	**48.1**	**32.7**	**19.2**
	**Sometimes in debt**	**11.6**	**44.2**	**44.2**
	**Always in debt**	**2.9**	**14.7**	**82.4**
	**Total (%)**	**24**	**31.8**	**44.2**

Note: Numbers in the cells are the percentages of responses within the rows of the table.

Chi-square analysis performed for cross tabulation, p-value <0.001. This illustrates a positive correlation between debt and lifestyle impacts from patient surveys.

#### Factors impacting on the success in the OST program

Survey participants were presented with six factors affecting the success of their OST, and asked to rank them from the one having the greatest impact (scored as 1) to the least impact (scored as 6). The factors were:

• Travel

• Dosing times

• The cost of dispensing fees

• Not having enough take-away doses

• Feeling judged

• Limited dosing venuesFigure 1 shows the distribution (numbers of responses) of the factors considered to have the greatest impact on success of the OST. Cost was clearly impacting on therapy as 64 participants (46.4%) ranked it as the factor most likely to influence their retention in the OST program.

**Figure 1:**
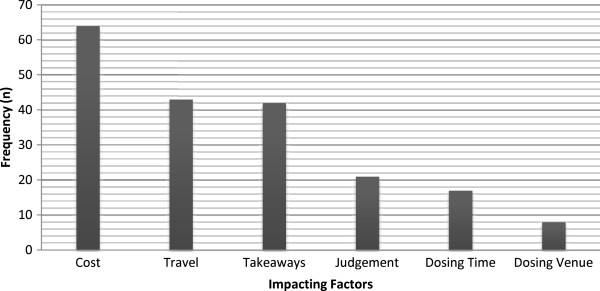
Survey data of factors impacting on OST patients’ success in the program (n = 138).

Logistic regression models were used to determine if any of the independent variables (cost impact, cost of therapy, relationship status, having children, employment status and the time spent in the OST program) influenced being in debt or having lifestyle difficulties. Table 3 summarises the results of these analyses, and shows that ‘Cost Impact’ significantly influenced both the occurrence of debt (57.7% vs 11.4%, p < 0.0001) and lifestyle difficulties (80.0% vs 33.3%, p = 0.0004). In addition, being in employment significantly (p = 0.0308) raised the odds of lifestyle difficulties (compared with those respondents not in employment). For these analyses, “Cost impact” was a binary variable (Yes/No) defined as “Yes” if cost was classified as either the most important or second most important factor affecting success of the OST program (ranked 1 or 2). These associations show that participants who identified that cost affected their success in the OST program were significantly more likely to incur debt and have lifestyle difficulties.

**Table 3:** Survey data factors influencing impact on debt and lifestyle: logistic regression analyses of these two dependent variables

**Dependent variable**	**Independent variable**	Number n/N (%)*	**Odds ratio**	**95%Confidence interval**	**p-value**
Being in debt	Cost impact				
	No	4/35 (11.4)	1 (reference)		
	Yes	30/52 (57.7)	10.6	3.3 to 34.3	<0.0001
Lifestyle difficulties	Cost impact				
	No	10/30 (33.3)	1 (reference)		
	Yes	48/60 (80.0)	6.6	2.3 to 18.9	0.0004
	Employed				
	No	8/22 (36.4)	1 (reference)		
	Yes	46/63 (73.0)	3.5	1.1 to 10.9	0.0308

*n/N shows the number of cases with a ‘significant impact’ response for each outcome, out of the total number in the row.

Logistic regression models used.

## Discussion

This study obtained opinions and feedback from patients and stakeholders about the impact of dispensing fees on patients’ debt, lifestyles and success or retention in the OST program. The stakeholders and OST patients both identified OST out-of-pocket dispensing fees as the main factor associated with OST non-compliance and early drop-out from the OST program. The survey data showed that dispensing fees impacted patients’ treatment in several ways including treatment compliance, debt accumulation and impact on lifestyle. All of these factors may increase the chance of a patient leaving the program. OST cost indeed received the highest rating in influencing retention in the OST program and there was a positive correlation between time on the OST program and success in terms of relapse prevention.

Treating a person with heroin or another opioid addiction is complex and the treatment process is multifaceted, requiring a holistic approach [28]. Seeking abstinence may not be the immediate goal but rather seeking normality and stability in the patient’s life [9,12]. The length of time a patient spends on OST is positively correlated with therapy success [19]. The first 12 months in OST is important as patients are at high risk of relapsing, are often financially unstable and vulnerable. It is therefore important to keep patients in the program during this initial stage. Retainment in the program in the initial phase of OST tends to increase the patient’s chance of obtaining long-term stability and abstinence from the addicted drug [26]. Patients often need to work on repairing relationships, treating other health issues (HIV [5,8], malnutrition [29], and mental health disorders [30]), and gain financial stability via a regular income source [22,28,31]. In order for patients to achieve these goals they require OST to treat the physical withdrawal symptoms caused by ceasing the use of the abused drug.

The findings from this study suggest that treatment is impacted negatively by out-of-pocket OST dispensing fees as patients often encounter financial pressures. As the study sample seems to be representative of the wider OST community in Western Australia [20] the findings should be similar for other OST patients in this state. This study also highlighted a number of reasons why a patient may drop out of an OST program, with the main reason being the cost of the dispensing fee. Previous studies indicated that financial incentives and rewards increase patient compliance [23,25,32]. This highlights the importance of financial assistance to improve patient retention rates in OST programs.

Survey data showed that OST patients’ lifestyles were impacted by the cost of the dispensing fees. Additionally, there was a correlation between participants who indicated that the fees impacted on their lifestyle and the accumulation of high personal debt. These findings show that the current charging practices of OST dispensing fees not only increase drop-out rates due to an increase in the relapse risk [33-35] and the inability to pay for doses, but also increase the risk of adverse health problems. Financial pressure from personal debt has been linked to an increased risk of substance abuse [36], mental health issues, depression, suicidal ideation, stress, and anxiety [36-38]. The prevalence of these health issues are high in drug addiction patients [39] and the added financial pressure from the dispensing fees are likely to exacerbate these conditions.

The World Health Organisation (WHO) states that healthcare systems should aim to provide equitable healthcare to all people. WHO defines health equity as “*the absence of unfair and avoidable or remediable differences in health services and outcomes among groups of people”*[40]. In 2000 WHO identified three areas of focus namely “*health, responsiveness and financing fairness*” [41]. Patients in OST programmes need to pay regular dispensing fees and they hence have extra health expenses. Although the PBS provides for the medicine itself to be entirely subsidised by the government, the fee associated with the dispensing of the medicine is not regulated and there is no financial assistance for this fee in Western Australia. Participants were of the opinion that OST dispensing fees should contribute towards the PBS Safety Net Scheme as is the case with patients who suffer from chronic conditions. Considering the current dispensing fees, Western Australian OST patients who obtain their OST medicines at community pharmacies pay on average $154 per month for their dosing, which is significantly more compared to patients paying for medicines for diabetes, smoking, and hypercholesterolemia. Although these conditions could also be the result of lifestyle choices, the dispensing of the medicines to treat these conditions contributes towards a patient’s Safety Net, which is not the case with OST medicines. Opioid dependence is therefore not considered at the same level as other medical conditions and OST patients are financially disadvantaged under the current PBS arrangements.

Interview participants indicated that patients are often in debt to pharmacies as a result of their inability to pay the dispensing fees. Pharmacy staff therefore need to follow-up with patients to recover the debt which adds to perceptions of stigma from staff as well as self-stigma by patients. It could be argued that pharmacists might be able to dose more patients and more pharmacies might be willing to participate in the OST program should the government sponsor the dispensing fees.

This study was limited by the small number of venues surveyed. In addition, the surveys relied on the accuracy of self-reported data. However, this method was deemed appropriate to obtain data from this vulnerable population. The study only involved OST patients within the Perth metropolitan area and therefore lacks country and rural patients’ perspectives. Furthermore, the study was conducted in Australia and the findings may not apply to settings outside Australia. Purposive sampling was used to locate key stakeholders, possibly limiting the generalisability of the interview findings. However, purposive sampling was deemed the most appropriate method of ensuring that stakeholders were familiar with the topic of discussion. The interview method can be subject to interview bias, and participants may lean towards responses which have greater social desirability. However, the potential for interviewer bias was minimised with the use of a standardised interview framework. Finally, this study was time-limited and conducted over a relatively short timeframe which reduced the scope of the study.

## Conclusion

This study provided insight into OST patients’ financial difficulties and the data suggest that out-of-pocket dispensing fees are likely to have a negative impact on Western Australian OST patients’ compliance with therapy, retention in the OST program and lifestyle. Government sponsorship of the OST dispensing fees should be considered as sponsorship would potentially increase the retention rates of income-poor OST program recipients.

## Competing interests

The authors declare that they have no competing interests.

## Authors’ contributions

HLH was responsible for conceptualisation and coordination of the project. AS and BP developed the interview guides and survey questions and conducted the stakeholder interviews. All authors conducted the focus group. AS transcribed the interviews and did the initial thematic analysis. BP performed the initial statistical analysis. Data interpretation and analysis were confirmed by HLH. All authors read and approved the final manuscript.
